# Change and variability in Antarctic coastal exposure, 1979–2020

**DOI:** 10.1038/s41467-022-28676-z

**Published:** 2022-03-04

**Authors:** P. A. Reid, R. A. Massom

**Affiliations:** 1 Australian Bureau of Meteorology, Hobart, Tas Australia; 2 Australian Antarctic Program Partnership, Institute for Marine and Antarctic Studies, University of Tasmania, Hobart, Tas Australia; 3 Australian Antarctic Division, Kingston, Tas Australia; 4 Australian Research Council, Australian Centre for Excellence in Antarctic Science, Institute for Marine and Antarctic Studies, University of Tasmania, Hobart, Australia

**Keywords:** Climate change, Cryospheric science

## Abstract

Increased exposure of Antarctica’s coastal environment to open ocean and waves due to loss of a protective sea-ice “buffer” has important ramifications for ice-shelf stability, coastal erosion, important ice-ocean-atmosphere interactions and shallow benthic ecosystems. Here, we introduce a climate and environmental metric based on the ongoing long-term satellite sea-ice concentration record, namely Coastal Exposure Length. This is a daily measure of change and variability in the length and incidence of Antarctic coastline lacking any protective sea-ice buffer offshore. For 1979–2020, ~50% of Antarctica’s ~17,850-km coastline had no sea ice offshore each summer, with minimal exposure in winter. Regional summer/maximum contributions vary from 45% (Amundsen-Bellingshausen seas) to 58% (Indian Ocean and Ross Sea), with circumpolar annual exposure ranging from 38% (2019) to 63% (1993). The annual maximum length of Antarctic coastal exposure decreased by ~30 km (~0.32%) per year for 1979–2020, composed of distinct regional and seasonal contributions.

## Abstract (web summary)

A new metric measuring the exposure of the Antarctic coastline to full open-ocean conditions reveals strong regional and seasonal change and variability occurred over the past four decades due to the loss and/or gain of an offshore sea-ice buffer.

## Introduction

Sea-ice concentration, extent and seasonality are well characterised from satellite data^1–3^ and form the basis of major assessments of polar sea-ice change and variability since the late-1970s^4,5^. An important additional climate and environmental factor neglected to date, however, is change and variability in the exposure of the Antarctic coastline to fully open-ocean conditions and including potentially destructive ocean waves, as a result of a loss or gain of a protective sea-ice “buffer”. Circumpolar patterns of Antarctic coastal exposure, and where and how they are varying and/or changing, have been unknown and unreported.

There is strong motivation to routinely and consistently measure and monitor change and variability in Antarctic coastal exposure in space and time. This is underpinned by an increasing urgency to identify, understand and accurately model the nature and drivers of environmental change around Antarctica’s vast and dynamic coastal periphery, and to more robustly predict both the coastal system’s likely response to changing climate and the effects of coastal change^5^. When and where present, sea ice that covers between ~3 and ~19 million km^2^ of the Southern Ocean surface, depending on season^3^, plays an important though poorly quantified role in buffering Antarctica’s vulnerable floating ice-sheet margins and coastal environment from potentially destructive ocean waves. This is due to the capacity of pack ice floes to efficiently damp and dissipate the energy of incoming waves before they can interact with coastal margins^6^. Because of this, any change in coastal exposure has important ramifications for: (i) the stability of certain ice shelves and iceberg calving rates^7,8^; (ii) coastal erosion^9^; and (iii) the structure of coastal benthic ecosystems^10^.

**Fig. 1:**
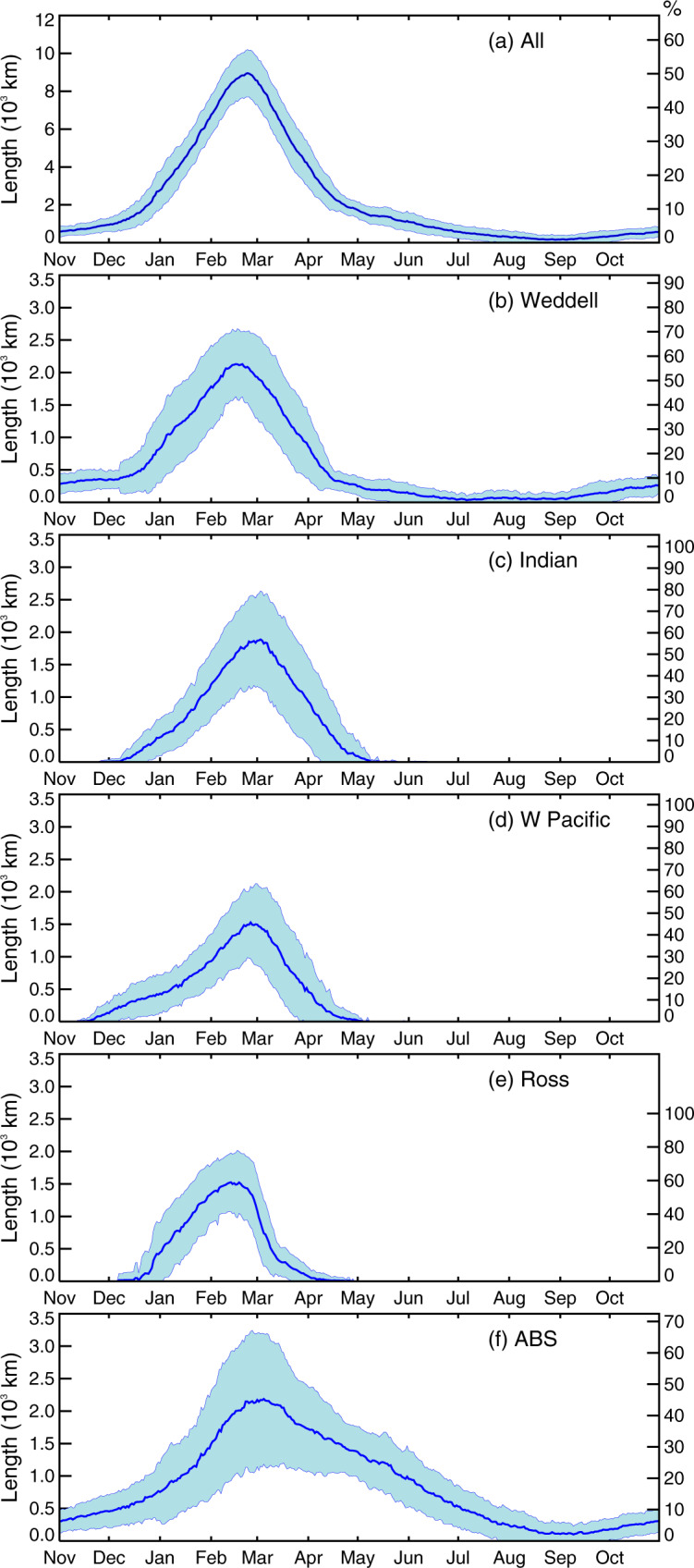
Plots of the average (1979–2020) coastal exposure length for each day (km). **a** Total Antarctic coastline, **b** Weddell Sea, **c** Indian Ocean, **d** W Pacific Ocean, **e** Ross Sea, and **f** ABS (Amundsen-Bellingshausen seas). Shaded blue lines represent ±1 standard deviation above and below the mean value.

Notably, recent work^8^ has implicated a regional change to increased sea ice-free conditions offshore from the western and north-eastern Antarctic Peninsula after the late-1980s in abrupt and catastrophic disintegration events of the Larsen A and B and Wilkins ice shelves since 1995^11,12^. The new paradigm^8^ is that this sea-ice loss exposed the ice-shelf fronts directly to the high-energy Southern Ocean, and enabled destructive ocean swell waves to reach and interact with the ice-shelf fronts over prolonged periods. This study^8^ showed that persistent wave-imposed flexure (cyclical bending) of the outer ice shelves then enlarged existing systems of rifts (through-cutting fractures) there to the point of extensive and spontaneous outer-margin calving in the form of multiple elongated icebergs. This triggered the large-scale rapid runaway disintegration of ice-shelf interior regions that had been substantially weakened by decades of warming, thinning, melt-related hydrofracture (surface meltwater enlargement of crevasses) and glaciological perturbations (see Fig. 1 and references in ref. ^8^). This rapid loss of ice-shelf buttressing capacity^13^ led in turn to an abrupt increase in the regional discharge of grounded ice-sheet mass into the ocean via feeder glaciers^14^, to contribute to an observed acceleration in mass loss from the Antarctic Ice Sheet^15,16^. In this way, a localised increased coastal exposure contributed to processes that led to sea-level rise^8^.

Here, we introduce a previously unconsidered climate and environmental metric, namely “coastal exposure length” (CEL). This is derived from the continuous daily satellite passive-microwave sea-ice concentration record dating back to 1979 (mapped to 25-km resolution), using a custom-developed algorithm (details given in the “Methods” section). Coastal exposure is here defined as a total lack of sea-ice coverage offshore from any given point on the coast i.e., uninterrupted exposure of the coastal margin to the open Southern Ocean as explained in detail in the Supplementary Section under “Analysis methods”. In this sense, the coastline is not taken to be “exposed” if there is any sea ice offshore even if the coast has open water adjacent to it in the form of leads or polynyas (areas of open water/thin ice that recur and persist in certain coastal locations around Antarctica^17^). “Sea ice” refers here to moving pack ice plus the narrower envelope of stationary consolidated fast ice that is affixed to extensive stretches of Antarctica’s coastline^18^.

First, we provide previously unknown baseline information on the climatological pattern of circum-Antarctic coastal exposure, in terms of both its spatial extent and duration and its regional and seasonal patterns and contrasts. We then highlight variability and trends in the exposure record, and reveal distinct and differing regional and seasonal contributions. Important wide-ranging implications of change in coastal exposure for ice-sheet processes and sea-level rise, coastal ecosystems and operations are evaluated in the “Discussion” section. The Methodology and a comparison with a similar Coastal Exposure Index (CEI) are included in the Supplementary material.

## Results

### Climatological patterns of Antarctic coastal exposure

**Fig. 2:**
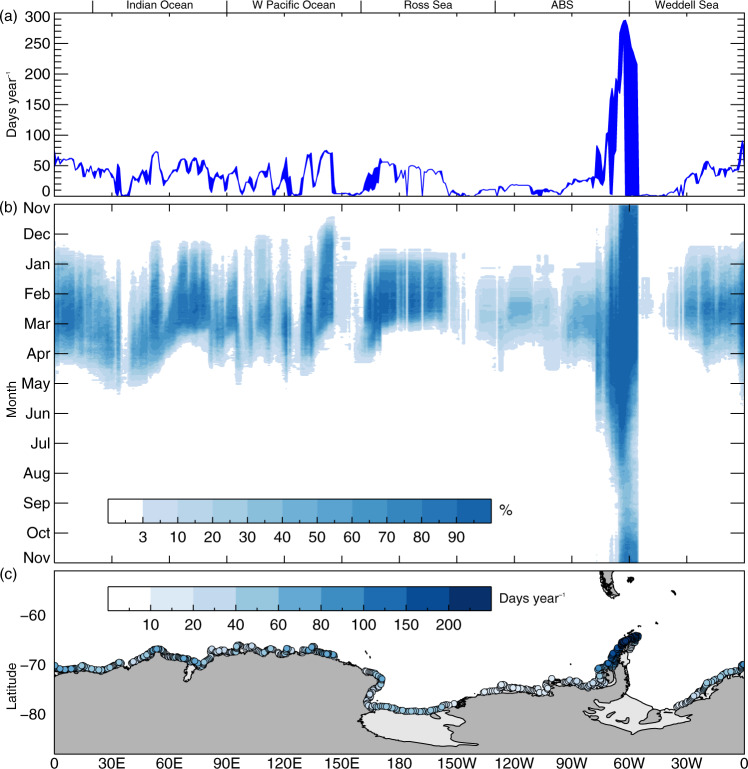
Climatology (1979–2020) of coastal exposure events based on coastal exposure length. **a** Annual exposure duration for each longitude (days year^−1^); **b** coastal exposure occurrence expressed as a percentage of frequency for each day and longitude (%); and **c** annual exposure for each grid point (days year^−1^). Shaded blue lines in (**a**) represent the variability (maximum and minimum) for each longitude. Grid points in (**c**) are arranged such that those with higher lengths of exposure are plotted over those with lower lengths.

The time series reveals distinctive large-scale patterns in the long-term daily means (for 1979–2020) of both the spatial length and timing of coastal exposure around Antarctica (Figs. 1 and 2). Average net circum-Antarctic CEL attains its maximum in late February (late austral summer), at ~8950 ± 1210 km or about 50% of the entire Antarctic coastal perimeter of ~17,850 km (Fig. 1a; Table 1). The climatological maximum in CEL sits within a roughly symmetrical annual cycle of 6 months increase in exposure and 6 months retreat. Particularly rapid rates of first increase then decrease in exposure length occur over roughly 2-month phases from mid-December through mid-February (summer) and March through April (autumn), respectively. The remaining 8 months are characterised by a long tail of low CEL (less than ~11% or ~2000 km) that tapers to a small late-winter climatological minimum CEL (of only ~33 km) in late August i.e., late winter (Fig. 1a). This climatological circum-Antarctic CEL cycle differs substantially from the mean annual cycle of overall sea-ice coverage (extent) in its timing and rates of seasonal change. The net sea-ice cycle is strongly asymmetric around advance and retreat seasons that run for ~7.5 months (mid-February through late September or late summer through early spring) and 4.5 months, respectively^3,19^.

**Table 1:** Statistics of coastal exposure length for the period 1979–2020.

	Total length of coastline (km)	Date of average maximum exposure (day of year)	Length of average maximum exposure (km)	Length of average maximum exposure as % of total coastline length	Length (km) and date of highest maximum exposure	Length (km) and date of lowest maximum exposure	Trend (km year^−1^) in annual maximum exposure
Total	17,850	23 Feb	8950	50	11,180 (3/3/1993)	6850 (28/2/2019)	−30
Weddell	3750	16–20 Feb	2130	57	3100 (28/2/1993)	1030 (9/2/2014)	−22
Indian	3300	3 March	1890	57	3180 (3/3/1987)	570 (several)	−22
W Pacific	3350	25 Feb	1530	46	2940 (several)	590 (several)	−18
Ross	2600	12–18 Feb	1520	58	2340 (several)	180 (several)	−6
ABS	4850	5 March	2,190	45	4610 (4/3/2010)	890 (several)	+36

The net annual CEL cycle (Fig. 1a) is composed of distinctly differing regional contributions (Fig. 1b–f—see Supplementary Analysis methods for the definition of regional sectors), with average annual maximum exposure of these regions expressed as a percentage of total coastline length. This ranges from ~45% for the Amundsen-Bellingshausen seas (ABS) to ~59% for the Ross Sea sector (Table 1). Of the five sectors, only the ABS and Weddell Sea have experienced some year-round coastal exposure during 1979–2020. Of these, the ABS is notable for both its broad annual exposure-decrease phase extending through winter and its relatively high interannual variability, with one standard deviation at peak annual exposure being >50% of the average value. The mean annual cycle in the Weddell Sea, on the other hand, most strongly resembles the net circum-Antarctic cycle.

In contrast and for the East Antarctic (Indian and W Pacific Ocean) and Ross Sea sectors, the mean annual window of coastal exposure is confined to late November through late April–early May (late austral spring through mid-autumn). Moreover, the mean annual exposure cycle across East Antarctica is characterised by a longer increase compared to decrease phase, by ~1 month in the Indian Ocean and ~1.5 months in the W Pacific. In the Ross Sea, CEL exhibits a particularly sharp seasonal decline of ~70% in only 2 weeks from late February through early March, after an annual peak that occurs ~2 weeks prior to those in the East Antarctic and ABS sectors but near-coincident with that in the Weddell Sea.

Strong regional dependence is also apparent in the mean duration of annual exposure and the timing of the annual exposure window as a function of longitude (Fig. 2)— together with complex intra-regional patterns of variability. Exposure duration covers a wide range, from low (<2 days) in parts of the W Weddell Sea, eastern Ross Sea and W Pacific Ocean sectors (as well as multiple more localised coastal tracts of the Indian and W Pacific oceans) to very high (~290 days) towards the northern tip of the Antarctic Peninsula (Fig. 2a). Elsewhere, mean annual exposure duration is generally ≤50 days, with a broader zone of shorter (<20 days) exposure across the ABS sector that ramps up eastwards towards the Antarctic Peninsula. Broadly speaking, zonally extensive regions of low and zero exposure duration (Fig. 2a) and frequency of occurrence (Fig. 2b) centred on ~155°E, 110°W and 50°W align with well-documented areas of perennial sea-ice coverage offshore^19^. Other regions of low exposure are centred on ~145°W and also within protected bays at ~37°E (Lützow-Holm Bay) and ~127°E (Porpoise Bay).

Regional contrasts and intra-regional variability are also a key feature of the mean timings of annual exposure onset and cessation, the resultant seasonal window of exposure duration and the frequency of exposure occurrence as a function of longitude (Fig. 2b). This again shows the Antarctic Peninsula to be a standout region in terms of the near year-round exposure along much of its western and north-eastern flanks. Elsewhere, the mean timing of annual exposure onset varies by about 2 months—from mid-November in the East Antarctic sector at ~140–150°E (George V Land coast) to mid-January in western parts of the Indian Ocean and Amundsen Sea. The anomalously early exposure onset in George V Land is likely linked to the recurrent Mertz Glacier Polynya, which has been shown in previous work^20^ to play an important role in driving an unusually early and rapid annual meltback of sea ice to the coast in that region. In autumn, the timing of mean annual exposure cessation again varies regionally over ~2 months, from early March (Ross Sea) to early May (Enderby Land in the western Indian Ocean).

The East and West Antarctic sectors in fact differ substantially in terms of their respective zonal patterns of mean coastal exposure occurrence timings (Fig. 2b). For East Antarctica from ~0° to 160°E, mean exposure-window duration is relatively uniform at about 4 ± 0.5 months, but displays a broad wave-like configuration that comprises alternating zones of apparent eastward and westward propagation over time for both exposure onset and subsequent cessation. For example, the exposure window propagates from west to east adjacent to the eastern limb of the Weddell Gyre (0–40°E) over a >1 month period, but this pattern reverses along much of the adjacent Indian Ocean coast.

From the Indian Ocean, the broad wave-like pattern in mean CEL duration transitions eastwards into a zone of more variable and generally lower frequency of occurrence across the W Pacific Ocean. This changes to a narrower (~3-month) and more uniform coastal-exposure window across the central Ross Sea. Moving eastwards into the ABS sector, the exposure window is initially fairly narrow (~2–3 months), occurs later and is more variable (than the Ross Sea), and also has a substantially lower frequency of occurrence. Exposure duration broadens towards the Antarctic Peninsula, where there is a high frequency of exposure occurrence throughout the year. This is in stark contrast to the adjacent area of zero exposure occurrence in the W/SW Weddell Sea due to perennial sea-ice coverage there. Finally, and from the central Weddell Sea through the Greenwich Meridian, the exposure occurrence window broadens back to ~4 to >5 months, with the timing of exposure onset remaining uniform but cessation timing increasing with distance towards the meridian. These distinct zonal patterns are further depicted in a map of the annual-mean duration of coastal exposure for 1979–2020 for all coastal-adjoining pixels (Fig. 2c).

### Variability in Antarctic coastal exposure

**Fig. 3:**
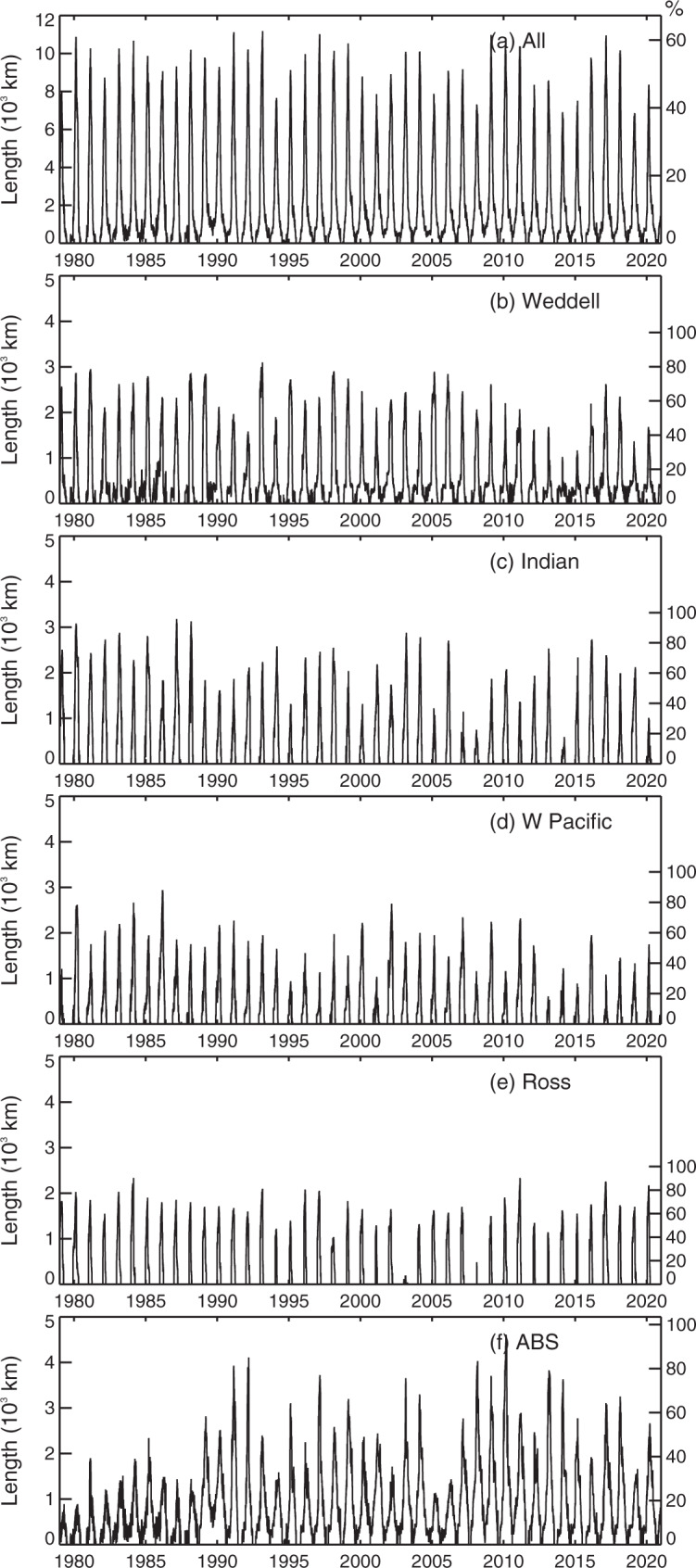
Time series plots of daily coastal exposure length (in km) for 1979–2020. **a** Total Antarctic coastline, **b** Weddell Sea, **c** Indian Ocean, **d** W Pacific Ocean, **e** Ross Sea, and **f** ABS.

**Fig. 4:**
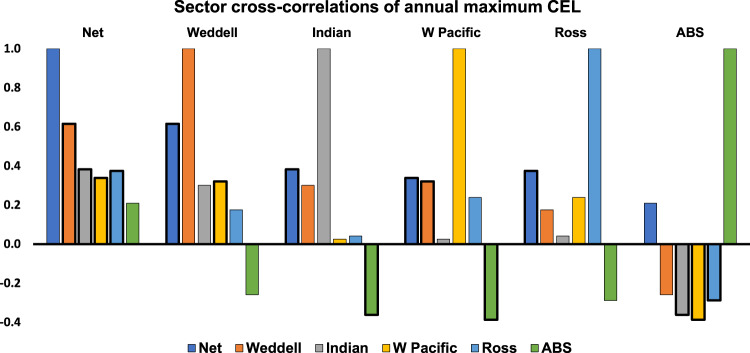
Trends sector cross-correlations of annual maximum based on coastal exposure length. Statistically significant values (>95% confidence) are shown in bold.

Previously unseen patterns of variability are also revealed in the daily time series of net overall CEL for 1979–2020, with annual maximum exposure ranging from 38% (~6850 km) in 2019 to 63% (11,180 km) in 1993 and interannual variability spanning apparent quasi 4- to 6-year cycles (Fig. 3a). This net circum-Antarctic picture is again made up of distinct regional contributions (Fig. 3b–f), with each of the five sectors displaying different and at times contrasting patterns of interannual variability in both annual maximum and (for the Weddell and ABS) annual minimum exposure. Net annual maximum CEL is most highly correlated to the Weddell Sea and least correlated to the ABS (Fig. 4 and Table 2), despite the ABS being the greatest regional contributor to total Antarctic coastline length. Annual maximum CEL for the Indian Ocean is most highly correlated to the Weddell Sea, and not correlated at all to other sectors. CEL in the ABS is anti-correlated to all other sectors, implying that processes determining coastal exposure in this sector differ from those elsewhere.

**Table 2:** Sector cross-correlations of annual maximum coastal exposure length for 1979–2020.

	Net	Weddell	Indian	W Pacific	Ross	ABS
Net	1.00	**0.62**	**0.38**	**0.34**	**0.37**	0.21
Weddell	**0.62**	1.00	0.30	**0.32**	0.18	−0.26
Indian	**0.38**	0.30	1.00	0.03	0.04	−**0.36**
W Pacific	**0.34**	**0.32**	0.03	1.00	0.24	−**0.39**
Ross	**0.37**	0.18	0.04	0.24	1.00	−0.29
ABS	0.21	−0.26	−**0.36**	−**0.39**	−0.29	1.00

Bold values indicate correlations with 95% confidence.

Particularly prominent is an abrupt change in the ABS sector from consistently low CEL values for that region (of largely <2000 km) prior to 1989 to generally higher values and greater interannual variability after that date (Fig. 3f). CEL in the ABS sector also generally trends upwards until 2010. Thereafter, there appears to be a trend towards less coastal exposure. In contrast, the earlier parts of the records for the Weddell and Ross seas and the Indian and W Pacific oceans (i.e., prior to 1989) are characterised by relatively high values of CEL (Fig. 3b–e). Very low values of summer-time (maximum) exposure occur in certain years after 2002, in: the Weddell Sea in 2014 (1020 km) and 2015 (1160 km, both versus a mean of 2130 km [Table 1]); the Indian Ocean in 2008 (750 km) and 2014 (570 km vs. 1920 km); the W Pacific Ocean in 2013 (590 km vs. 1670 km); the Ross Sea in 2003 (180 km) and 2008 (480 km vs. 1520 km); and the ABS sector in 2005 (1130 km vs. 2190 km). Moreover, the circumpolar time series contains anomalous years of relatively extensive annual CEL minima (Fig. 3a), with the net mean-winter CEL remaining positive only in the winters of 1983 (99 km), 1989 (particularly prominent at 392 km), 2001 (268 km), 2004 (318 km) and 2008 (294 km). This comprises positive mean-winter contributions from the Weddell Sea (only in 2004) and the ABS sector (in 1983, 1989 and 2008).

### Trends in Antarctic coastal exposure

**Fig. 5:**
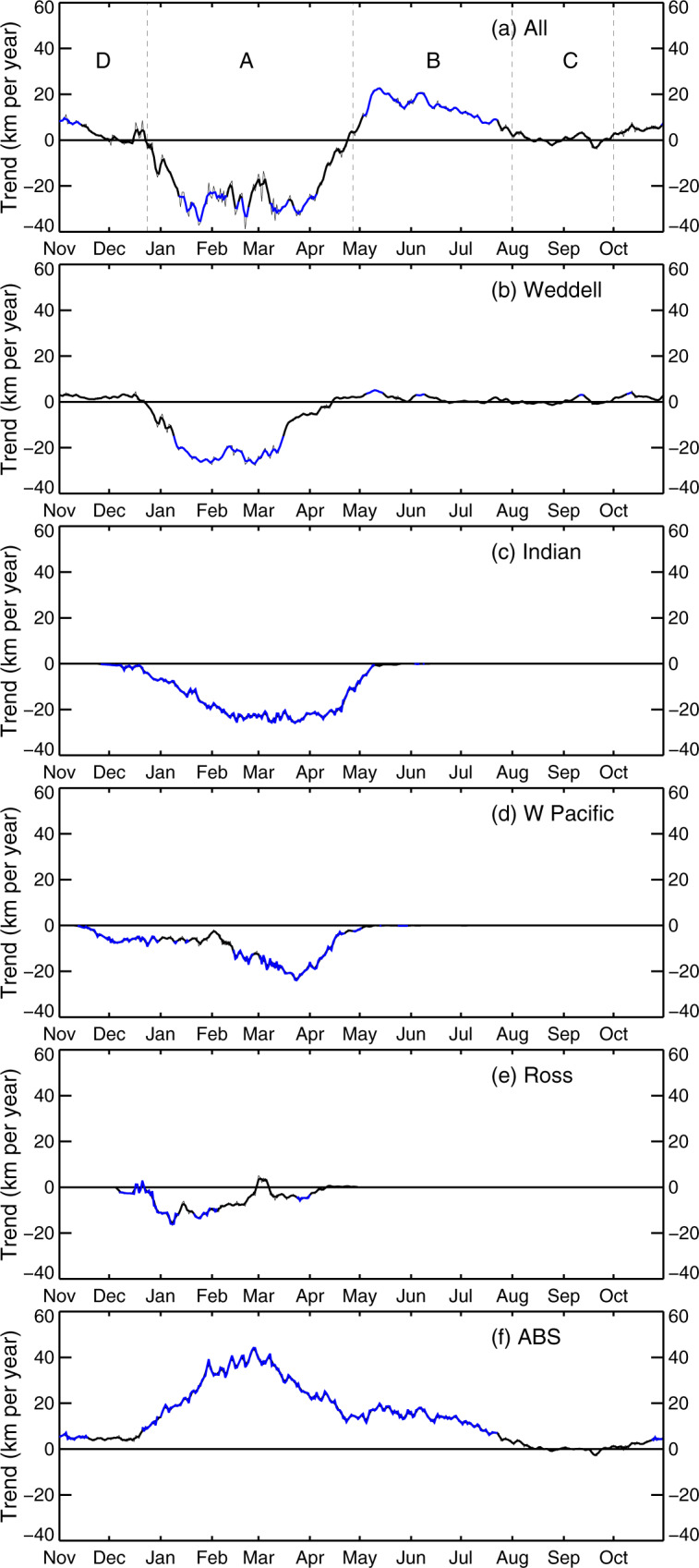
Plots of the trends (1979–2020) in coastal exposure length for each day (km year^−1^). **a** Total Antarctic coastline, **b** Weddell Sea, **c** Indian Ocean, **d** W Pacific Ocean, **e** Ross Sea, and **f** ABS. Statistically significant values (>95% confidence) are highlighted in blue. Thick black and blue lines are based on 3-day smoothing, while thin black lines are unsmoothed daily data. Periods A–D, as marked in (**a**), are mentioned in the text.

The annual maximum length of Antarctic coastline exposed to fully open-ocean conditions decreased by ~30 km (or ~0.32%) per year for 1979–2020, but this slight negative linear trend is composed of distinct regional and seasonal contributions. There are four standout seasonal contributions (as marked on Fig. 5a):

A. Late-December through April (early-austral summer through mid-autumn)— decreasing net CEL by as much as about 30–40 km year^−1^;
B. May through July (late-autumn through mid-winter)—a switch to increased CEL by up to about +20 km year^−1^;
C. August through September (late-winter through early-spring)—a 2-month period of near-zero change in net CEL; and
D. October–December (mid-spring through early-summer)—a return to increasing CEL, by up to approximately +10 km year^−1^.

There are again differing regional contributions (Fig. 5b–f) to the overall trend in net circum-Antarctic CEL (Fig. 5a), with the ABS being the only sector recording a significant positive (increasing) trend—apart from short and intermittent periods of small positive significant trends of up to +5 km year^−1^ in the Weddell Sea from mid-April through mid-December. The increasing trend in the ABS occurs throughout much of the year, apart from near-zero change in late winter-early spring (August–September), and peaks at +44 km year^−1^ in late February. Significant trends in the other four sectors are largely negative or close to zero, with the negative net trends of about −20 km year^−1^ being confined to late-spring through mid-autumn in the Weddell and Ross seas, and across the East Antarctic sectors.

**Fig. 6:**
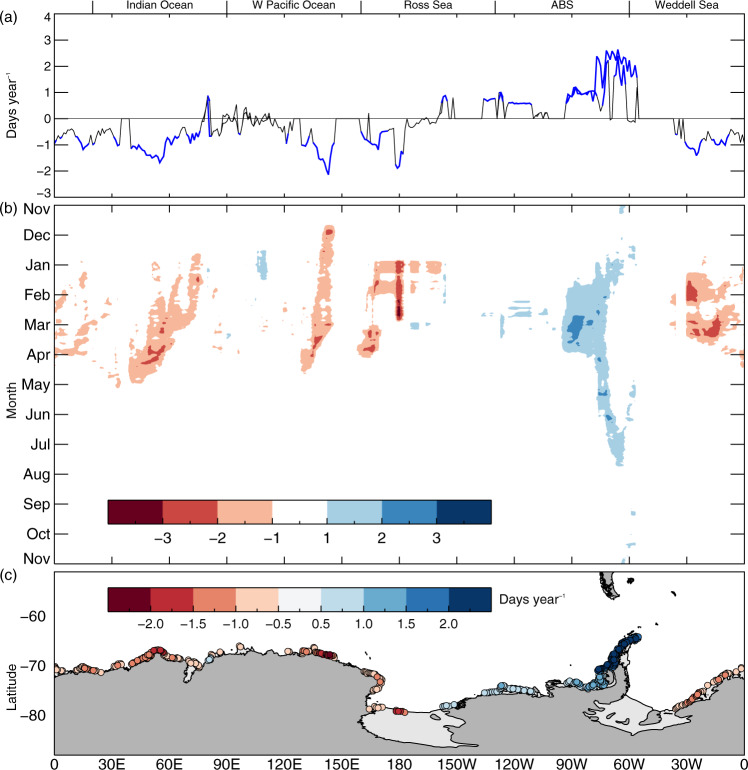
Trends (1979–2020) of annual coastal exposure events based on coastal exposure length. **a** Each longitude (days year^−1^), **b** each longitude and day (occurrence per decade), and **c** each coastal grid point (days year^−1^). Statistically significant values (>95% confidence) for (**a**) are highlighted in blue. Only statistically significant values (>95% confidence) are plotted on (**b**) and (**c**). For (**c**), grid points with higher magnitude of trends are plotted over those with lower magnitude.

Analysis of trends in coastal-exposure duration as a function of longitude and time (Fig. 6) again highlights the prominent trend to increasing exposure in the ABS sector— ranging from about approximately +0.5 to +3 days year^−1^. This occurs through much of the year, but most prominently in December through July and along the western Antarctic Peninsula, and is most extensive to the west in January through April (mid-summer through mid-autumn). Elsewhere, increasing exposure is confined to smaller coastal tracts and over shorter periods in summer in both the Amundsen Sea (during February) and the western part of the W Pacific Ocean (mid-December through mid-January).

In contrast, relatively extensive tracts of decreasing trend in coastal exposure (to −2 days year^−1^) occur in late-spring through mid-autumn across the eastern Weddell Sea (January through March), Indian Ocean (late-December through April), eastern part of the W Pacific Ocean (late-November through April) and western Ross Sea (late-December through March) (Fig. 6b). Intervening areas of zero trend in the eastern Ross and western Weddell seas (Fig. 6a) relate to the perennial presence of pack ice there^19^. The localised zero trend at ~140°E is associated with the build-up of persistent sea-ice coverage to the east of assemblages of grounded icebergs near the Mertz Glacier Tongue, and within the westward-flowing Antarctic Coastal Current^21^.

The negative trends in the Indian Ocean, eastern W Pacific Ocean and western Ross Sea sectors migrate westward by approximately 20° of longitude in autumn (March through May) (Fig. 6b). The positive trend in the West Antarctic Peninsula region undergoes a similar but earlier westward migration in summer (December through February), before reversing to an apparent eastward migration from autumn through winter (March through July). The reason for these regional trend propagation patterns is currently unknown. Figure 6c further shows that in some regions, such as around the West Ice Shelf (~85°E), there are trends of opposite sign at adjacent grid points—a complexity not resolved in the Hovmöller-style diagram of Fig. 6b.

## Discussion

The CEL introduced in this study represents an important and previously unavailable large-scale climate variable and metric against which to detect, gauge, monitor and assess physical change and variability in Antarctica’s unique and immensely influential yet vulnerable coastal environment on a daily basis from 1987 (and every other day from 1979 to July 1987). The ongoing CEL time series is also highly complementary and value adds to the existing time series of sea-ice concentration, extent, area and seasonality, as derived from the same satellite data source dating back to 1978^1,3^. As such, it will continue to provide important parallel information on the state of the Antarctic coastal environment over coming decades.

This analysis has revealed previously unknown climatological patterns as well as spatial and temporal variability and change in coastal exposure to fully open-ocean conditions around Antarctica. It shows the net annual exposure cycle to be: (1) symmetrical around a late-February maximum with an annual minimum in late August (which differs markedly from the asymmetric annual sea-ice advance and retreat cycle^3^); and (2) made up of highly-distinct regional contributions in terms of exposure spatial extent, timings of annual onset and cessation, and duration and frequency of exposure occurrence. Contrasting patterns of long-term mean exposure in East versus West Antarctica occur within an austral summer-through-autumn window, while the northern Antarctic Peninsula is the only region where exposure occurs year round.

In large part, the trend for 1979–2020 is towards negative coastal exposure, by as much as −20 km year^−1^ and −2 days year^−1^, and in austral summer through autumn and across all sectors apart from the Amundsen-Bellingshausen seas. There, spatial and temporal trends are positive and up to +44 km year^−1^ and +3 days year^−1^, respectively, and extend from mid-spring through winter. The ABS region has previously been identified as a “hot spot” for decreasing annual sea-ice duration^1^, a different climate and environmental metric to CEL.

There are multiple wide-ranging implications of both positive and negative changes in coastal exposure shown here, for current and future epochs—with each warranting further investigation beyond this study. For example and based on recent findings^7,8^, it is speculated that increased exposure could potentially diminish the stability, size and buttressing capacity of certain remaining ice shelves, depending on additional ice shelf-specific glaciological factors and regional setting—by increasing interaction of their outer margins with potentially destructive ocean swells leading to increased iceberg carving^8^. Ice-shelf areal loss would in turn increase the rate of the Antarctic Ice Sheet’s contribution to sea-level rise^14^.

It is further proposed that change to increased incidence of coastal exposure and resultant iceberg production would have substantial impact on coastal and offshore ecosystems, and in both positive and negative ways. On the positive, for example, icebergs produced by increased wave-induced ice sheet calving can substantially seed the upper ocean in their vicinity with freshwater and nutrients, to enhance ocean primary production and support high concentrations of krill, marine life and seabirds to distances of a few kilometres out from each berg^22^. On the negative, an increase in the number of icebergs would also increase their destructive scouring of nearshore benthic ecosystems (to depths of about 450 m)^23^. Change to increased coastal exposure could also increase wave-generated turbulence, the deposition of biogenic material, and light availability for photosynthesis and heating. Shallow coastal benthic ecosystems are particularly vulnerable to such environmental change as a result of sea ice loss^10,24^.

Furthermore, knowledge of temporal and spatial characteristics of coastal exposure can contribute to our understanding of complex non-linear interactions and feedbacks between coastal sea ice and ocean processes. For example, changes in coastal configuration^25^ and the distribution of grounded icebergs likely impact coastal currents, which may change coastal exposure. This may in turn further affect ice-shelf stability and morphology and Antarctic coastal evolution^26,27^. Another avenue of further research is to determine the drivers of the observed patterns of change and variability in CEL. This includes investigation of the response of coastal exposure to the observed higher variability in overall Antarctic sea-ice extent since 2012^3,28^.

It is hoped that the findings presented here will, in concert with emerging studies highlighting the regional effects of changing coastal exposure^8^, motivate representation of change in circumpolar Antarctic CEL in improved ice-sheet models and next-generation Earth system models. It is anticipated that this will contribute to the development of more accurate and robust predictive capability regarding the fate of the vulnerable Antarctic coastal environment and remaining ice shelves under a changing climate, which is crucial to projecting Antarctica’s future contribution to sea-level rise. Such an advance will also provide additional and previously unavailable information towards coastal-exposure forecasting in support of safe and efficient Antarctic logistical operations and shipping^29^. Such developments are timely and crucial, given that the magnitude and effects of coastal exposure are likely to be heightened in future by predicted increases in wind speed and wave height in the high-latitude Southern Ocean^30,31^.

## Supplementary information

Supplementary Information

## Data Availability

Daily sea ice concentration data^32^ can be obtained from the NASA National Snow and Ice Data Center (NSIDC) Distributed Active Archive Center (DAAC), directly available from: https://nsidc.org/data/seaice_index. Data for producing all Coastal Exposure Index and Coastal Exposure Index figures are available from: https://data.aad.gov.au/metadata/records/AAS_4116_4528_Coastal_Exposure.

## Code availability

Interactive Data Language (IDL) code for calculating CEL from daily sea ice concentration data can be obtained from: https://data.aad.gov.au/metadata/records/AAS_4116_4528_Coastal_Exposure.
