# Supplementary material for: Change and variability in Antarctic coastal exposure, 1979–2020
Source: Nat Commun. 2022 Mar 4;13:1164. doi: 10.1038/s41467-022-28676-z (PMC8897499; doi:10.1038/s41467-022-28676-z)
Supplement: Supplementary file 1 — Supplementary Information [file 41467_2022_28676_MOESM1_ESM.pdf]

Change and Variability in Antarctic Coastal Exposure, 1979-2020 – Supplementary

Reid, P. A.[1,2*], and R. A. Massom[2,3,4]

[1]Australian Bureau of Meteorology, Hobart, Tasmania, Australia

[2]Australian Antarctic Program Partnership, Institute for Marine and Antarctic Studies, University of Tasmania, Hobart, Tasmania, Australia

[3]Australian Antarctic Division, Kingston, Tasmania, Australia

[4]Australian Research Council, Australian Centre for Excellence in Antarctic Science, Institute for Marine and Antarctic Studies, University of Tasmania

* Corresponding author address: Phillip Reid, Australian Bureau of Meteorology, GPO Box 727, Hobart, Tasmania 7001, Australia. E-mail: phillip.reid@bom.gov.au

Supplementary

Data

The main CEL method, and a subsidiary Coastal Exposure Index or CEI (both described below), are based on daily sea-ice concentration products for the period 1979 through 2020. These products are derived from the multi-satellite passive-microwave brightness temperature time series using the NASA Team algorithm[1], mapped at 25 km x 25 km resolution and obtained from the NASA National Snow and Ice Data Center (NSIDC) Distributed Active Archive Center (DAAC). Both algorithms are designed to be adaptable for different resolution data. Complete coverage of the entire Antarctic coastal and sea-ice zones is obtained on a daily basis, except for 1979-July 1987 (once every two days). Missing single-day values during this period are interpolated from the adjoining day's sea-ice concentration values. Averages and climatologies are based on the period 1979-2020, unless otherwise stated. The continental land mask used (gsfc_25s.msk) is also obtained from NSIDC, and includes ice shelves (the seaward extremities of which are taken here to be coastline). Coastline grid points are defined from the continental land mask as any ocean grid point that has land/ice sheet adjacent to it. The South Shetland Islands (at a longitude of ~60°W) and Mill Island (~101°E) are neglected in the calculations of coastal exposure since they permanently block the calculation of CEI from the continental edge.

Analysis methods

For this study, we developed and evaluated two new but different algorithms for quantifying and monitoring coastal exposure: the Coastal Exposure Index (CEI) and a more complex Coastal Exposure Length (CEL) method. The CEI technique is based on the detection of sea ice presence/absence radially out (northwards) from the coastline along each meridian (at one degree longitudinal spacing), following masking of the ice sheet (e.g., Supplementary Fig. 1a). As such, the CEI is simply defined as the number of longitudes with no sea ice (threshold set to < 15% following convention) to the north of the continent, and hence runs from zero to 360.

While the relatively-simple CEI algorithm provides reasonable broad-scale assessment of coastal exposure (see the daily example in Supplementary Fig. 1a) and is computationally inexpensive, it fails to categorise certain coastal areas as exposed when they clearly are. As a result, it tends to underestimate total exposure in terms of both its physical length and duration. Specifically, the application of CEI is limited where the (exposed) coastline being assessed: (1) is N-S trending (e.g., the Antarctic Peninsula); (2) has a zonal-trending promontory of land/ice sheet to its north; and/or (3) is semi-enclosed by a zonally-extending sea ice tongue offshore. An example of the latter, across the Amundsen Sea at ~105-115°W, is given in Supplementary Fig. 1a; such features tend to be common though sporadic and ephemeral, particularly during the late annual sea-ice retreat season (December-January).

For this reason, the more sophisticated CEL algorithm was developed, and the output from this is used in the main analysis as it does not have the disadvantages of the CEI. Here, CEL is defined as the length (in kms) of the Antarctic coastal perimeter with no adjacent sea ice anywhere offshore (i.e. total exposure of the coast to the open Southern Ocean with no intervening sea ice), but excluding coastal polynyas (recurrent areas of persistent open water/thin ice enclosed by sea ice[2]) e.g. Supplementary Fig. 1b. By this method, we use the land mask to determine if each coastal grid point has an immediately-adjacent ocean grid point that is ice-free (i.e. has a sea-ice concentration of <15%). If this criterion is met, then a nearest (adjoining) neighbour-testing technique is used to determine whether that ocean grid point is exposed in some way to the wider open ocean or is bound by neighbouring sea ice offshore. If any of the neighbouring grid points are classified as "exposed", or if the total area of neighbouring ice-free grid points exceeds an arbitrary cut-off of 500,000 km^2, then that coastal grid point is classified as "exposed". Otherwise, the grid point and all sea-ice-free neighbouring grid points are deemed to be bounded by sea ice and are classified as a coastal polynya. The

length of individual exposed coastal grid points is estimated by taking the square root of the respective pixel area. The length of coastal exposure, either regionally or net circum-Antarctic, is then simply the sum of the length of exposed coastal grid points.

Although the CEL algorithm is computationally more expensive than the CEI technique (especially where large coastal polynyas are present), it provides more complete coverage of coastal exposure at greater detail. Moreover, and as a by-product, the CEL method readily detects, maps and monitors regions of low (<15%) sea ice concentration that are bounded by higher-concentration sea ice, i.e. coastal polynyas, as shown in orange at ~80°W and ~90°E in Supplementary Fig. 1b. This additional information on polynya size and distribution (not provided here as it is outside the scope of this study) is also important from a coastal-exposure perspective in that Antarctic coastal polynyas make a regionally-important contribution to the seasonal melt-back of sea ice to the coast each austral summer[3]. The CEL method also has wider geographic applicability i.e. around the Arctic sea-ice regions, and a follow-up analysis of change and variability in Arctic coastal exposure is planned.

Within the analysis, delineation of regional sectors follows the widely-used protocol for sea-ice analysis[4]. Austral spring is taken to be September through November, summer is December through February, autumn is March through May, and winter is June through August. Leap years are plotted on Figs. 1 and 5, along with Supplementary Figs. 2 and 6, however values for the 29th February in these figures are linearly interpolated from the 28th February to the 1st March. Trend plots within Fig. 5 and Supplementary Fig. 6 have smoothing applied, based on a moving 3-day non-weighted smoothing. Throughout the main text, trends and associated significance are calculated using the IDL (Interactive Data Language) trend subroutine. Error estimates associated with mean values are presented as one standard deviation of the entire time series. Figure 4 and Supplementary Fig 5 were plotted using Microsoft Excel. All other figures were produced using IDL.

Strictly speaking, the presence/absence of coastal sea ice in this study refers to both moving pack ice and stationary fast ice that is attached (in certain places[5]) to the coast and icebergs grounded on near-coastal shoals <450 m deep[6]. However, the coarse spatial resolution of the passive microwave-based indices precludes specific detection of fast ice or its accurate distinction from pack ice - as Antarctic fast ice where present typically occurs in a relatively narrow coastal band[5]. Fine-scale analysis of coastal exposure involving lack/loss of fast ice

therefore requires finer-scale satellite data and analysis techniques, which are beyond the scope of this broad-scale study. Having said this, there is some correspondence between loss/lack of pack ice and loss/lack of adjacent sea ice, due to the susceptibility of coastal fast ice to breakup by ocean swells in the absence of a protective pack ice "buffer"[7,8].

Brief comparison of CEL versus CEI

Here, we carry out a brief comparative assessment of the CEL and CEI methods for our Antarctic application, by replicating the CEL results shown in the main analysis in Figs. 1 to 6 using the CEI method i.e., Supplementary Figs. 2 to 7. This comparison confirms the wider applicability of the CEL method. Notably, the frequency of occurrence of CEL (Fig. 2a) is slightly higher than that of CEI (Supplementary Fig. 3a), and CEL exposure has an earlier onset than CEI-derived exposure at the same longitude (Fig. 2b versus Supplementary Fig. 3b), although timings of exposure cessation are very similar except in the Ross Sea sector. These differences are further highlighted by comparison of the statistics of CEL versus CEI results shown in Supplementary Tables 1 and 2, respectively. Generally speaking, however, the magnitude, seasonality and regional distribution of both climatology and trends obtained from CEL (Figs. 1 to 6) and CEI (Supplementary Figs. 2 to 7) are quite similar in their overall patterns. In summary, CEL is better suited to more detailed analyses of coastal exposure requiring higher accuracy (and hence it was chosen for this study), while CEI is more suited to the assessment and analysis of model output regarding current and future climate scenarios.

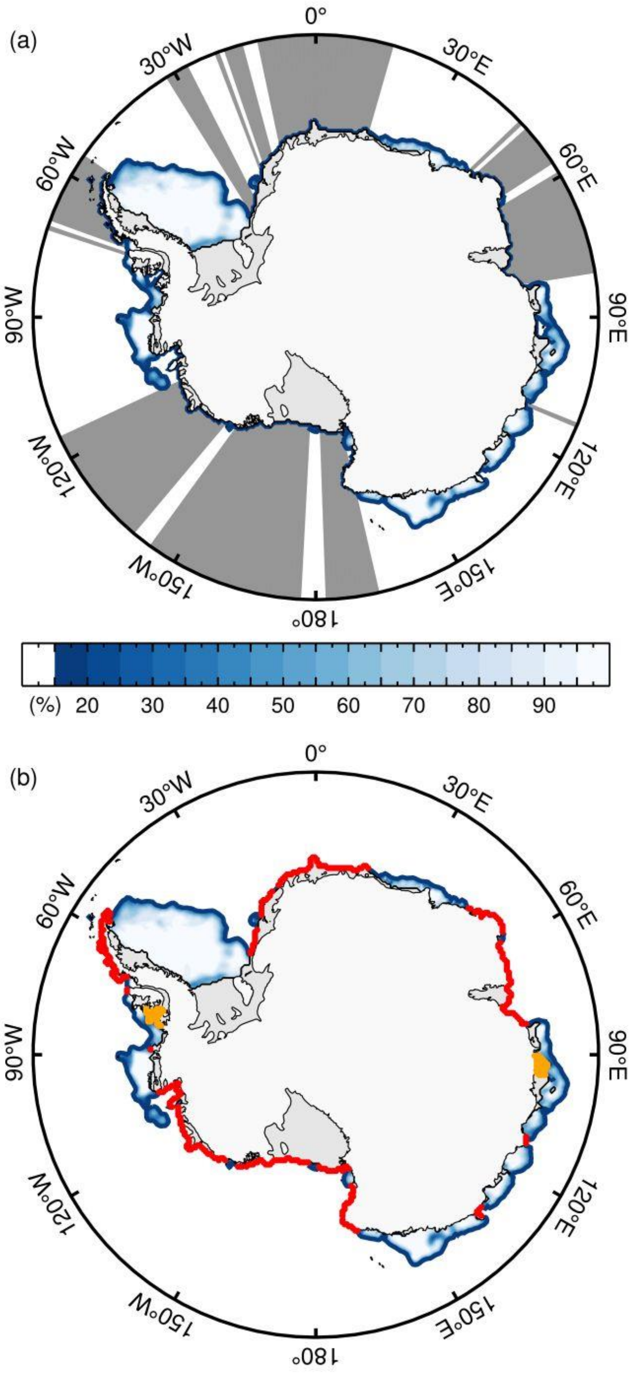

Supplementary Fig. 1 Examples of the two methods of representing Antarctic coastal exposure, from 14 February 2017. (a) CEI, where the dark grey shows each longitude of coastal exposure; and, (b) CEL, where red dots represent each coastal pixel unbounded by any sea ice and orange dots represent low-concentration bounded pixels. The blue gradient on each map represents the sea-ice concentration (%) for that day.

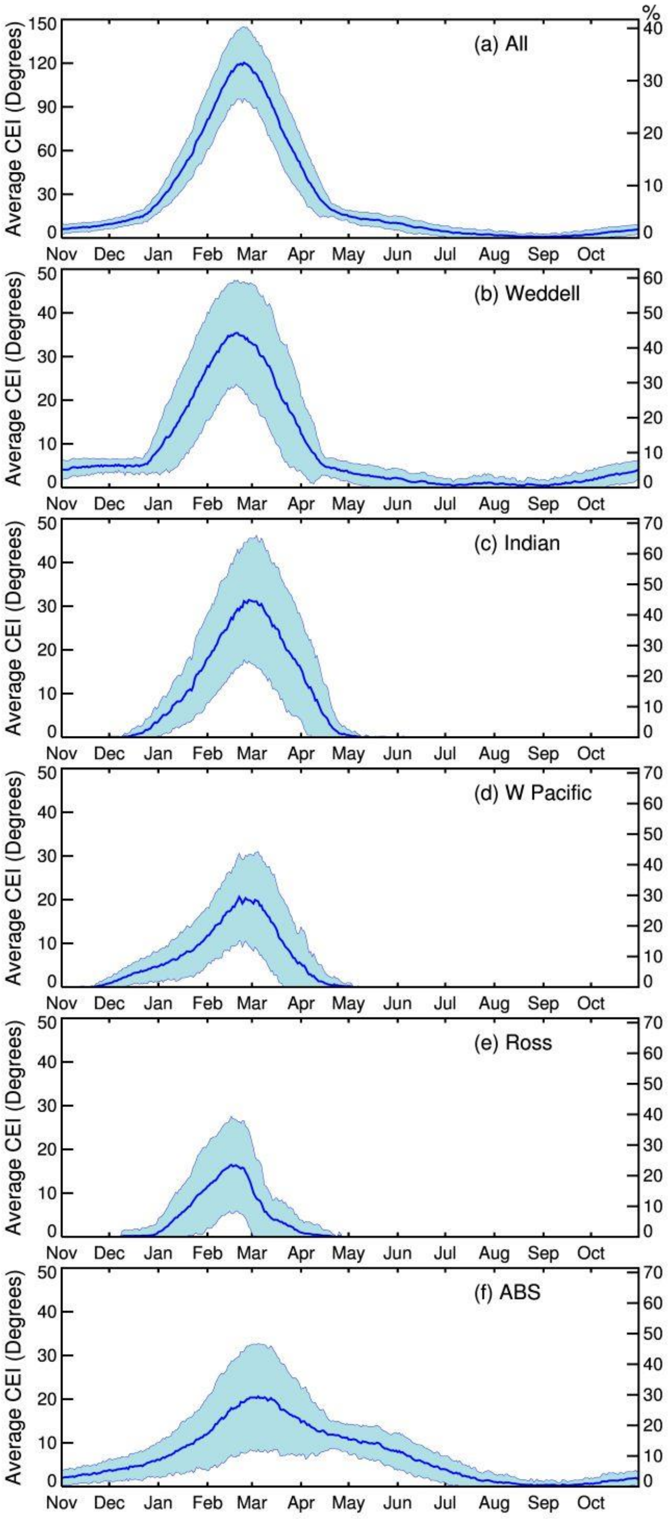

Supplementary Fig. 2 Plots of the average (1979-2020) coastal exposure index for each day (degrees) using the CEI method. (a) Total Antarctic coastline, (b) Weddell Sea, (c) Indian Ocean, (d) W Pacific Ocean, (e) Ross Sea, and (f) ABS. Shaded blue lines represent ±1 standard deviation above and below the mean value.

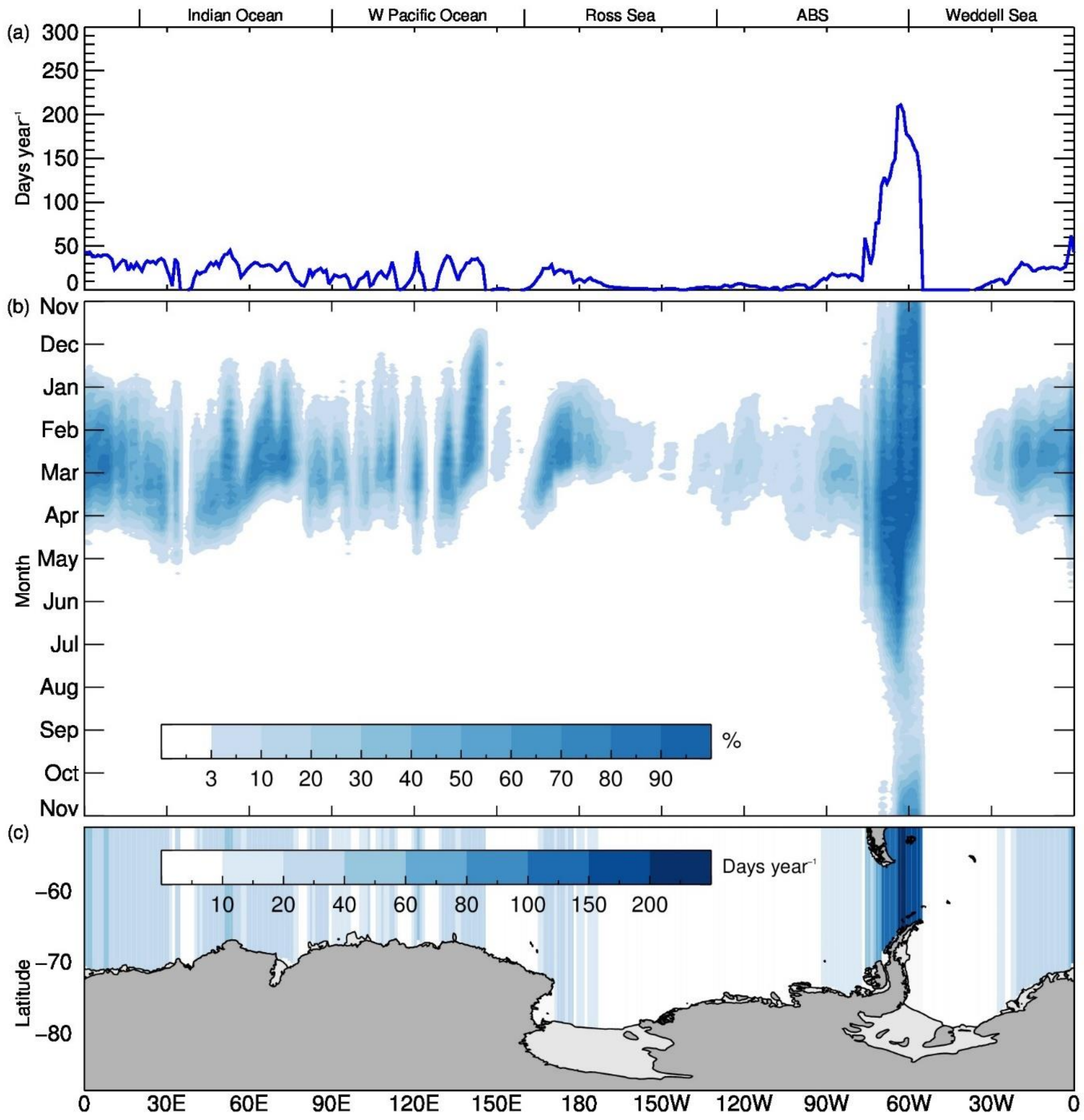

Supplementary Fig. 3 Climatology (1979-2020) of CEI. (a) Each longitude (average days year^{-1}), (b) coastal exposure occurrence expressed as a percentage of frequency for each day and longitude (%), and (c) each grid point (days year^{-1}).

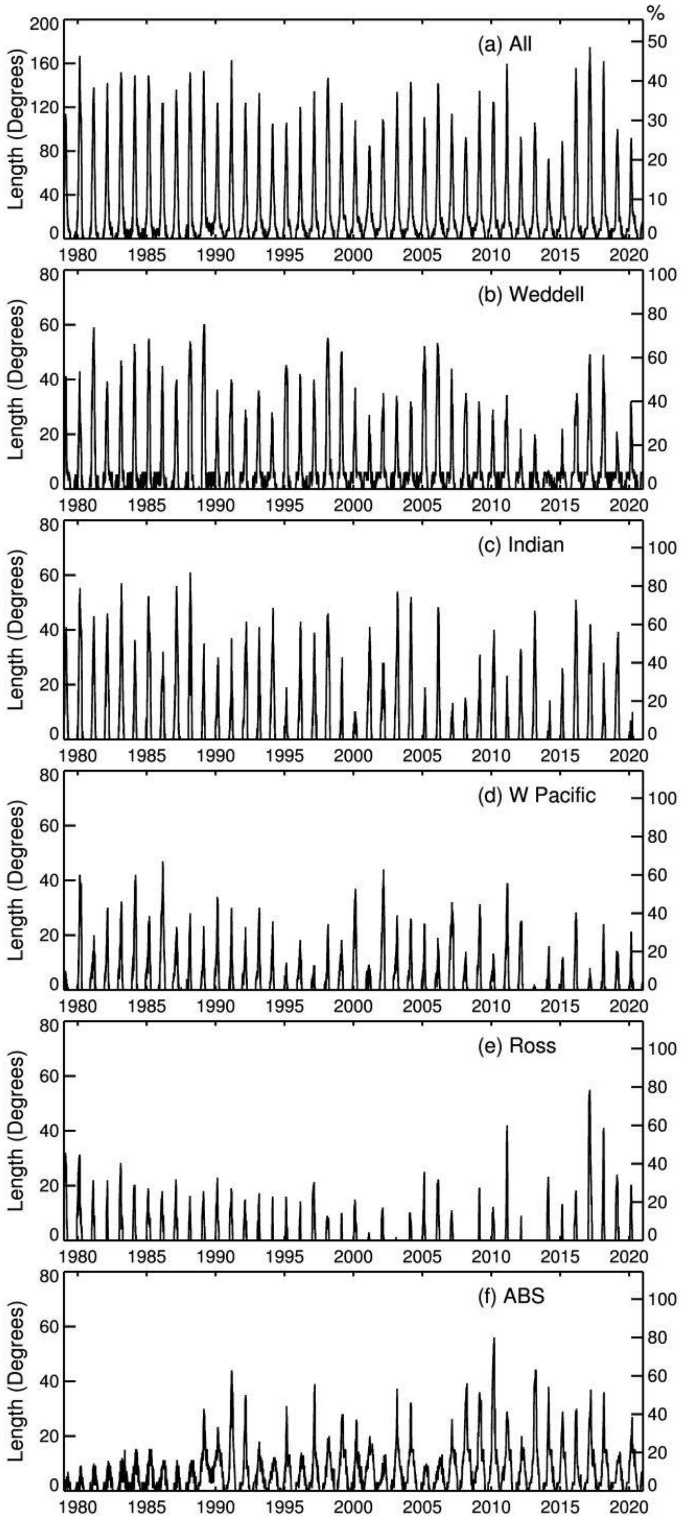

Supplementary Fig. 4 Time series plots of daily coastal exposure index for 1979-2020. (a) Total Antarctic coastline, (b) Weddell Sea, (c) Indian Ocean, (d) W Pacific Ocean, (e) Ross Sea, and (f) ABS.

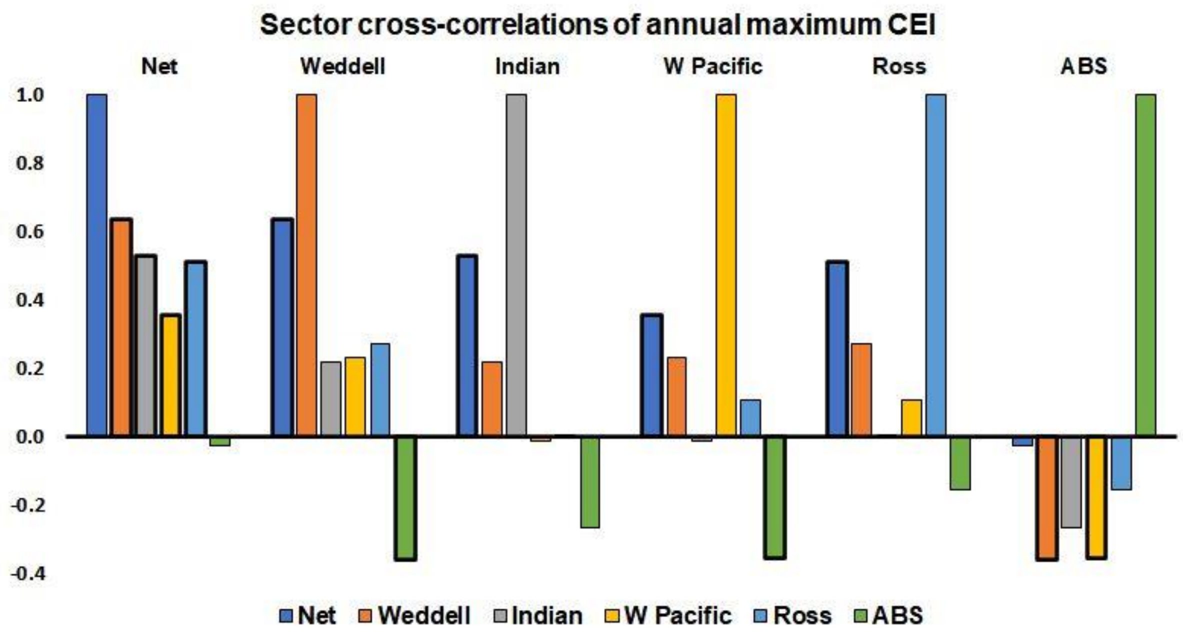

Supplementary Fig. 5 Sector cross-correlations of annual maximum coastal exposure index. Statistically-significant values (>95% confidence) are shown as bold.

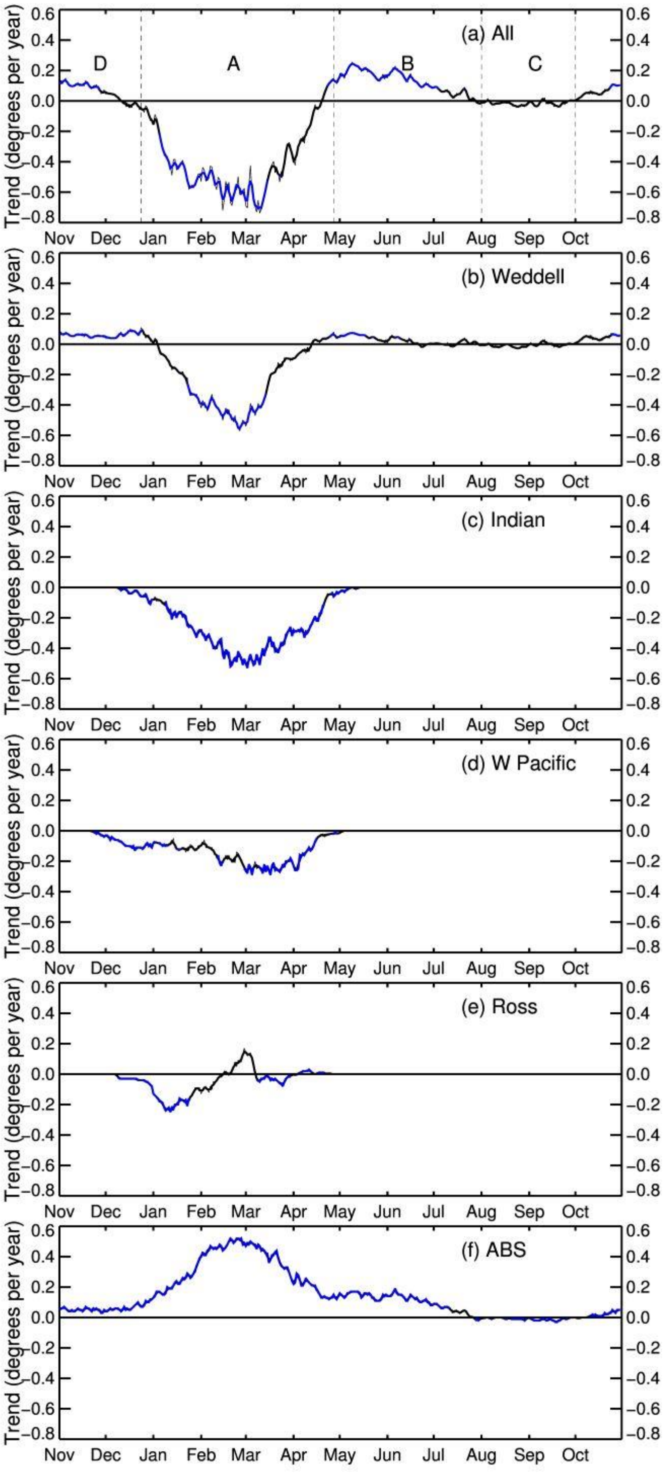

Supplementary Fig. 6 Plots of the trends (1979-2020) in CEI for each day (degrees year^{-1}). (a) Total Antarctic coastline, (b) Weddell Sea, (c) Indian Ocean, (d) W Pacific Ocean, (e) Ross Sea, and (f) ABS. Statistically-significant values (>95% confidence) are highlighted in blue. Thick black and blue lines are based on 3-day smoothing, while thin black lines are unsmoothed daily data. Seasons A, B, C and D, as marked in (a), are discussed in the text.

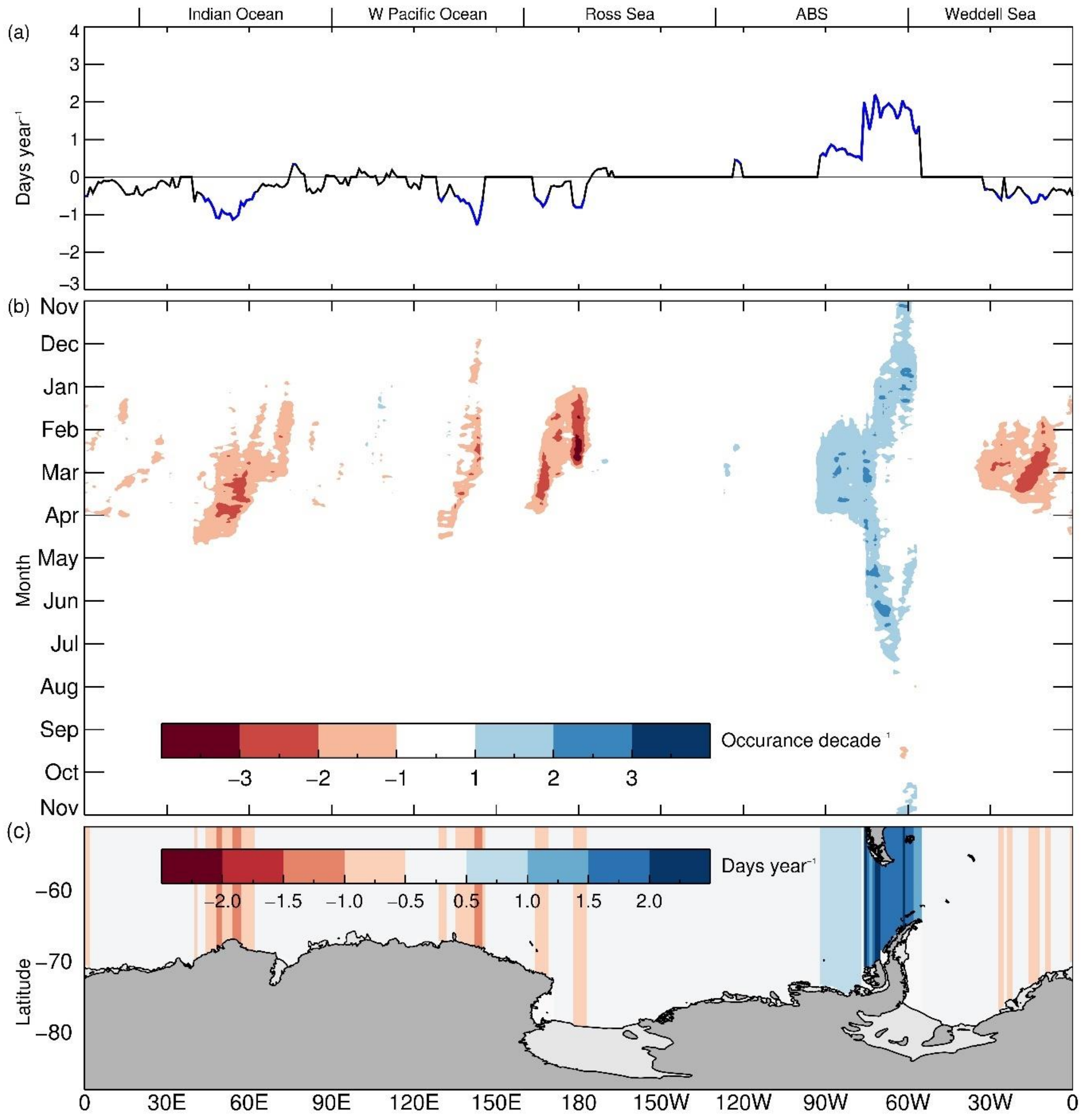

Supplementary Fig. 7 Trends (1979-2020) of annual coastal exposure index for CEI. (a) Each longitude (days year^{-1}); (b) each longitude and day (occurrence per decade); and (c) each grid point (days year^{-1}). Statistically-significant values (>95% confidence) for (a) are in blue. Only statistically-significant values (>95% confidence) are plotted on (b) and (c). For (c), grid points with higher magnitude of trends are plotted over those with lower magnitude.

	Length of coastline (degrees)	Date of average maximum exposure (day of year)	Length of average maximum exposure (degrees)	Length of average maximum exposure as percentage of length of coastline (%)	Length (degrees) and date of highest maximum exposure	Length (degrees) and date of lowest maximum exposure	Trend (degrees $year^{-1}$) in annual maximum exposure
Total	360	24 Feb	120	33	175 (24/2/2017)	73 (several)	-0.67
Weddell	80	14-24 Feb	35	44	60 (several)	7 (20/2/2014)	-0.48
Indian	70	24 Feb - 7 March	31	49	61 (4/3/1988)	10 (several)	-0.48
W Pacific	70	21 Feb	21	30	47 (5/3/1986)	2 (several)	-0.29
Ross	70	13-24 Feb	16	23	55 (24/2/2017)	0 (several)	-0.01
ABS	70	4 March	21	30	56 (18/3/2010)	7 (15/4/1979)	+0.53

Supplementary Table 1 Statistics of coastal exposure index (CEI) for the period 1979-2020.

	Net	Weddell	Indian	W Pacific	Ross	ABS
Net	1.00	**0.64**	**0.53**	**0.36**	**0.51**	-0.03
Weddell	**0.64**	1.00	0.22	0.23	0.27	**-0.36**
Indian	**0.53**	0.22	1.00	-0.01	0.01	-0.27
W Pacific	**0.36**	0.23	-0.01	1.00	0.11	**-0.36**
Ross	**0.51**	0.27	0.01	0.11	1.00	-0.16
ABS	-0.03	**-0.36**	-0.27	**-0.36**	-0.16	1.00

Supplementary Table 2 Sector cross-correlations of annual maximum coastal exposure index for 1979-2020. Bold values indicate correlations with 95% confidence.

References

1. Cavalieri, D. J., Parkinson, C. L., Gloersen, P. & Zwally, H. J. Sea Ice Concentrations from Nimbus-7 SMMR and DMSP SSM/I-SSMIS Passive Microwave Data, Version 1. *Boulder, Colorado USA. NASA National Snow and Ice Data Center Distributed Active Archive Center* (1996). doi:https://doi.org/10.5067/8GQ8LZQVL0VL

2. Barber, D. G. & Massom, R. A. Chapter 1 The Role of Sea Ice in Arctic and Antarctic Polynyas. in *Polynyas: Windows to the World* (eds. Smith, W. O. & Barber, D. G. B. T.-E. O. S.) **74**, 1–54 (Elsevier, 2007).

3. Massom, R. A. *et al.* An anomalous late-season change in the regional sea ice regime in the vicinity of the Mertz Glacier Polynya, East Antarctica. *J. Geophys. Res. Ocean.* **108**, (2003).

4. Zwally, H. J., Comiso, J. C., Parkinson, C. L., Cavalieri, D. J. & Gloersen, P. Variability of Antarctic sea ice 1979–1998. *J. Geophys. Res. Ocean.* **107**, 9–19 (2002).

5. Fraser, A. *et al.* High-resolution mapping of circum-Antarctic landfast sea ice distribution, 2000–2018. *Earth Syst. Sci. Data Discuss.* **15**, 1–18 (2020).

6. Massom, R. A. *et al.* Fast ice distribution in Adélie Land, East Antarctica: interannual variability and implications for emperor penguins Aptenodytes forsteri. *Mar. Ecol. Prog. Ser.* **374**, 243–257 (2009).

7. Langhorne, P. J., Squire, V. A., Fox, C. & Haskell, T. G. Lifetime estimation for a land-fast ice sheet subjected to ocean swell. *Ann. Glaciol.* **33**, 333–338 (2001).

8. Massom, R. A. *et al.* Antarctic ice shelf disintegration triggered by sea ice loss and ocean swell. *Nature* **558**, 383–389 (2018).
